# Novel Insights into the Diversity of Catabolic Metabolism from Ten Haloarchaeal Genomes

**DOI:** 10.1371/journal.pone.0020237

**Published:** 2011-05-25

**Authors:** Iain Anderson, Carmen Scheuner, Markus Göker, Kostas Mavromatis, Sean D. Hooper, Iris Porat, Hans-Peter Klenk, Natalia Ivanova, Nikos Kyrpides

**Affiliations:** 1 Genome Biology Program, Department of Energy Joint Genome Institute, Walnut Creek, California, United States of America; 2 Deutsche Sammlung von Mikroorganismen und Zellkulturen, Braunschweig, Germany; 3 INEOS Bio, Fayetteville, Arkansas, United States of America; University of Hyderabad, India

## Abstract

**Background:**

The extremely halophilic archaea are present worldwide in saline environments and have important biotechnological applications. Ten complete genomes of haloarchaea are now available, providing an opportunity for comparative analysis.

**Methodology/Principal Findings:**

We report here the comparative analysis of five newly sequenced haloarchaeal genomes with five previously published ones. Whole genome trees based on protein sequences provide strong support for deep relationships between the ten organisms. Using a soft clustering approach, we identified 887 protein clusters present in all halophiles. Of these core clusters, 112 are not found in any other archaea and therefore constitute the haloarchaeal signature. Four of the halophiles were isolated from water, and four were isolated from soil or sediment. Although there are few habitat-specific clusters, the soil/sediment halophiles tend to have greater capacity for polysaccharide degradation, siderophore synthesis, and cell wall modification. *Halorhabdus utahensis* and *Haloterrigena turkmenica* encode over forty glycosyl hydrolases each, and may be capable of breaking down naturally occurring complex carbohydrates. *H. utahensis* is specialized for growth on carbohydrates and has few amino acid degradation pathways. It uses the non-oxidative pentose phosphate pathway instead of the oxidative pathway, giving it more flexibility in the metabolism of pentoses.

**Conclusions:**

These new genomes expand our understanding of haloarchaeal catabolic pathways, providing a basis for further experimental analysis, especially with regard to carbohydrate metabolism. Halophilic glycosyl hydrolases for use in biofuel production are more likely to be found in halophiles isolated from soil or sediment.

## Introduction

The organisms in the euryarchaeal order Halobacteriales are generally extreme halophiles requiring at least 1.5 M salt and growing optimally at 3.5–4.5 M salt [1], although some have recently been found to grow at lower salt concentrations [2], [3]. The haloarchaea are found in the water and sediment of salt lakes and salterns, and also in saline soils. Their mechanism of adaptation to high salinity involves the accumulation of molar concentrations of KCl in the cytoplasm and the production of proteins with a higher number of negative charges than in other organisms. Bacteria of the order Halanaerobiales and the genus *Salinibacter* also accumulate KCl internally [4]. Other halophilic organisms accumulate compatible solutes such as glycerol or glycine betaine to counter high external salt concentrations, but this is energetically more costly than accumulation of KCl [5].

The haloarchaea are heterotrophs, growing with amino acids and/or carbohydrates as carbon and energy sources. They are either aerobic or facultatively anaerobic using various electron acceptors [1]. Glycerol is a particularly important nutrient for haloarchaea as it is produced by eukaryotic algae in high-salt environments [6]. Dihydroxyacetone, produced by *Salinibacter ruber*, can also be present at high concentrations [7]. While some haloarchaea are capable of growth on a wide range of compounds, others are very limited in their metabolism. The most extreme example of this is *Haloquadratum walsbyi*, which has only been found to grow well on pyruvate or dihydroxyacetone [7], [8].

Six haloarchaeal genomes have been sequenced previously, and analyses of the genomes have been published for *Halobacterium salinarum* NRC-1 [9], *Halobacterium salinarum* R1 [10], *Haloarcula marismortui* [11], *Natronomonas pharaonis* [12], *Haloquadratum walsbyi* [13], and *Haloferax volcanii* [14]. Since the two *H. salinarum* genomes are very similar, we included only strain NRC-1 in the analysis. Here we present an analysis of these five genomes along with five new genomes, four of which (*Halogeometricum borinquense*, *Halomicrobium mukohataei*, *Halorhabdus utahensis* and *Haloterrigena turkmenica*) were sequenced as part of the Genomic Encyclopedia of Bacteria and Archaea (GEBA) project [15]. The remaining genome, *Halorubrum lacusprofundi*, was sequenced as part of a Joint Genome Institute Community Sequencing Program project to sequence diverse archaeal genomes. With this work we are doubling the amount of genomic information from these extremophilic organisms and derive novel information and conclusions about the breadth of their metabolic capabilities.

## Methods

### Genome sequencing

Sequencing and annotation of *H. borinquense*, *H. mukohataei*, *H. utahensis*, and *H. turkmenica* have been previously described [16]–[19]. A publication describing the sequencing and annotation of *H. lacusprofundi* is in preparation. Genome sequences are available from GenBank. Genome analysis was carried out within the Integrated Microbial Genomes Expert Review (IMG-ER) system [20].

### Sequence data used in phylogenetic analysis

Protein sequences from 19 *Halobacteria* and outgroup (*Methanomicrobia*) genomes were retrieved from the IMG website (http://img.jgi.doe.gov/) or from NCBI (*Methanocella paludicola SANAE*; NC_013665). *Methanomicrobia* appear as sister group of *Halobacteriaceae* in a recent comprehensive 16S rRNA tree [21]. Accession numbers for the genomes used in this study are listed in Table S5.

### Orthologs and alignments

All-against-all protein BLAST was performed using mpiBLAST version 1.5 (http://www.mpiblast.org/), a parallel implementation of NCBI BLAST [22], using soft masking instead of complexity filtering. To determine orthologs, BLAST e-values were transformed using our own re-implementation of the OrthoMCL algorithm [23] in conjunction with MCL version 08-312 (http://micans.org/mcl/) using an inflation parameter of 3.0 (slightly deviating from the default, 2.0, thus yielding slightly more clusters). The transformation of the e-values corrects for genome-specific biases as, e.g., caused by a GC bias [23]; after transformation, the BLAST results are reduced to the reciprocal best hits for each pair of genomes, which are then clustered using the MCL algorithm. OrthoMCL clusters containing inparalogs were reduced by selecting the most ‘central’ of several sequences from the same genome, that is, the sequence with the highest sum of within-cluster BLAST scores. The reduced OrthoMCL clusters were aligned using MUSCLE version 3.7 under default settings [24]. The program scan_orphanerrs from the RASCAL package version 1.3.4 [25] was applied to detect orphan sequences (overall poorly aligned genes) within the alignments. After removal of orphan sequences (if present), poorly aligned columns and divergent regions were eliminated with GBLOCKS version 0.91b [26] using a minimum block length of two amino acids and allowing gap positions in all sequences. Each OrthoMCL cluster was also assigned to a COG category [27] using a majority-rule approach. Above-mentioned parameter settings for ortholog determination and alignment filtering had previously been optimized using a genome-scale dataset for *Actinobacteria* type strains (unpublished data). During this optimization, it also turned out that modifying the program parameters had comparatively little influence on the phylogenetic outcome.

### Phylogenetic inference

Filtered OrthoMCL cluster alignments comprising at least four sequences were concatenated to form a supermatrix for phylogenetic analysis. Maximum likelihood (ML) phylogenetic trees were inferred from the supermatrix with the Pthreads-parallelized RAxML package [28] version 7.2.5, applying fast bootstrapping with subsequent search for the best tree [29], the autoMRE bootstopping criterion [30] and the LG model of amino acid evolution [31] in conjunction with gamma-distributed substitution rates [32] and empirical amino acid frequencies (F). Among all amino acid models implemented in RAxML (except GTR, which was rejected for performance reasons), LGF was the empirically preferred one, as it produced the highest likelihood if optimized on a RAxML parsimony starting tree. Tree searches under the maximum parsimony (MP) criterion were conducted with PAUP* version 4b10 [33] using 100 rounds of random sequence addition and subsequent TBR branch swapping, saving no more than 10 best trees per round and collapsing potential zero-length branches during tree search. MP bootstrap support was calculated with PAUP* using 1,000 replicates with 10 rounds of heuristic search per replicate.

### Assessing incongruence between gene trees and species tree

After reducing the OrthoMCL cluster alignments to ingroup sequences, congruence between gene trees and the species tree was assessed by calculating partitioned Bremer support (PBS) [34], [35] for each OrthoMCL cluster using the newick.tcl script [36] in conjunction with PAUP*. The Bremer support value for a certain bipartition (split) in the tree topology is the difference between the score (number of steps) of the best-known MP tree in which this split does not occur (forced using a converse constraint) and the score of the unconstrained best-known MP tree. While the total Bremer support must be positive, PBS values may be positive, zero, or negative, indicating that the OrthoMCL cluster supports the split, contains no information, or prefers another topology, respectively [34]. As the total PBS (summed over all branches) for each cluster positively correlated with both its number of genes and its number of informative characters, the residuals from a linear regression with these two independent variables were determined. The residuals were used to determine their correlation with the COG categories and classes [27] and the clusters most in conflict with the species tree (i.e., those with the most negative total PBS).

### Spectral clustering

Protein sequences for the ten halophiles were downloaded from IMG-ER [20]. We applied a spectral clustering procedure [37], [38] for discriminating between groups of homologous proteins. The proteins are represented as nodes in a connected undirected graph with edges that carry weights based on node-to-node similarity according to the protein identity. The clustering procedure is analogous to a random walk of a particle moving on this graph from one node to another. In each node the particle moves to another node based on the probabilities corresponding to the weights of the edges. The amount of time the particle spends in a given subgraph will determine whether this is indeed a cluster of its own or not. After infinite time, the distinction between these subgraphs will become more clear, and to model this we calculated the normalized transition matrix at equilibrium. The eigenvalues of the transition matrix provide a measure of how distinct (or entwined) subgraphs are in relation to each other. An eigenvalue (or strength of partition) of 1 suggests a complete distinction between two subgraphs, while an eigenvalue of 0 suggests that no further partitions can be made. The eigenvectors are ordered by their eigenvalues in descending order, and the graph is successively partitioned while the second eigenvalue is greater than 0.8. As a result, a graph is partitioned into one or more subgraphs until the distinction between subgraphs becomes less clear. In this work, the choice of 0.8 as a cutoff suggests a balance between partitioning only fully non-homologous proteins and allowing proteins with weak similarities to be separated into subgraphs.

### Proteases and glycosyl hydrolases

The heat maps and clustering of secreted proteases and glycosyl hydrolases were generated using hierarchical clustering with the program Cluster [39] and visualized with TreeView (http://rana.lbl.gov/EisenSoftware.htm). Glycosyl hydrolases were identified based on Pfam domains. Proteases were obtained from MEROPS [40]. Non-peptidase homologues were eliminated from the analysis. Signal peptides were identified with SignalP [41].

## Results

### Orthologs and alignments

The number of aligned and filtered OrthoMCL clusters containing at least four entries (i.e., genes from distinct genomes) was 3,853, their length ranging from 22 to 1650 amino acids (262.542 on average). The concatenated supermatrix thus comprised 1,011,575 columns, including 708,296 variable and 353,936 parsimony-informative characters.

### Phylogenetic inference

**Figure 1:**
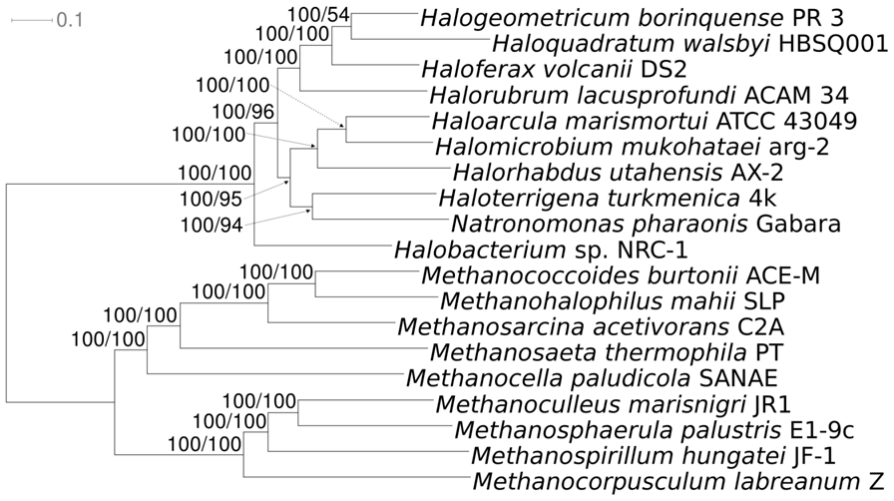
Maximum likelihood (ML) phylogenetic tree inferred from the 3,853-gene supermatrix. The branches are scaled in terms of the expected number of substitutions per site. Numbers above branches are support values from ML (left) and maximum parsimony (MP; right) bootstrapping. The tree was rooted with the *Methanomicrobia* genomes included in the sample. The topology of the single best MP tree was identical to the one depicted here.

The ML phylogeny inferred from the concatenated gene alignments is shown in Figure 1 (species tree) together with ML and MP bootstrap support values. The final highest log likelihood obtained was −12,342,390.79, whereas the single best MP tree (excluding uninformative sites) had a score of 1,409,187. ML and MP topologies were identical. Support was maximum (100%) for all branches under ML, and maximum for all but four branches under MP; only a single branch entirely lacked support under MP. As only one genome per genus was included in the sample, there is no taxonomic subdivision of *Halobacteriaceae* to compare the tree with. However, outgroup taxonomy was well recovered, the tree showing the monophyly of *Methanomicrobiales*, *Methanosarcinales*, and *Methanosarcinaceae*, each of which were represented with at least two genomes.

### Incongruence between gene trees and species tree

After reducing the dataset to the ingroup taxa and to the OrthoMCL clusters present in at least four ingroup genomes, total PBS per OrthoMCL cluster ranged between 142 and −219 (average: 4.941, standard deviation: 19.588, median: 1, MAD: 8.896). These data are plotted against the number of parsimony-informative characters in supplementary Figure S1. Within a total of 2,891 OrthoMCL clusters, 1,506 genes showed overall positive support and 764 showed overall negative support. Trees inferred from the five clusters least congruent with the species tree are depicted in Figure S2. They are uniformly characterized by high bootstrap support for groupings in conflict with the species tree estimate. Total PBS values per cluster vary between the COG categories (which could be assigned to 2,213 clusters; see Figure S1); on average, COGs related to information storage and processing display higher PBS than those associated with metabolism or cellular processes and signaling (Table S1); but individual COG categories may differ from this general trend (Table S1).

### Core clusters

We used a spectral clustering method to generate gene clusters from the haloarchaeal genomes. There were 887 core clusters, those found in all of the haloarchaeal genomes, and these accounted for 40% to 50% of the genes in each genome (Table 1). As expected, the core clusters contain genes involved in basic cellular processes such as transcription, translation, DNA replication, DNA repair, RNA modification, protein modification, and protein secretion (Table S2). The core clusters also include many genes involved in biosynthesis of essential metabolites – amino acids, purines and pyrimidines, lipids, and cofactors. This is somewhat unexpected as the haloarchaea are heterotrophs, but they appear to be relatively self-sufficient in being able to make most essential metabolites. Biosynthetic pathways in the haloarchaea have recently been reviewed [42], so we will not go into more detail here. The number of genes in each genome belonging to all clusters ranges from 78% to 88% (Table 1), showing that 12% to 22% of the genes in each genome have no hits to genes in the other halophile genomes.

**Table 1:** Cluster distribution.

Genome	protein-coding genes	genes in core clusters	genes in all clusters	clusters in all except this genome
*H. salinarum*	2675	1337 (50.0%)	2342 (87.6%)	61
*H. marismortui*	4348	1705 (39.2%)	3770 (86.7%)	2
*N. pharaonis*	2843	1400 (49.2%)	2444 (86.0%)	20
*H. walsbyi*	2861	1407 (49.2%)	2422 (84.7%)	78
*H. volcanii*	4063	1679 (41.3%)	3488 (85.8%)	4
*H. lacusprofundi*	3665	1556 (42.5%)	3135 (85.5%)	14
*H. borinquense*	3937	1639 (41.6%)	3311 (84.1%)	1
*H. mukohataei*	3416	1510 (44.2%)	2999 (87.8%)	6
*H. utahensis*	3027	1407 (46.5%)	2524 (83.4%)	64
*H. turkmenica*	5287	2028 (38.4%)	4152 (78.5%)	10

### Signature clusters

We also identified signature gene clusters, those that are shared by all haloarchaea but are not found in any other archaea. There are 112 of these clusters (Table S3), 89 of which contain proteins with completely unknown function. Of the clusters with a predicted function, two are protein kinases related to the *Bacillus subtilis* PrkA protein. These two kinase genes are adjacent to each other on the chromosome in all haloarchaeal genomes and are always found with two other genes: one with a domain of unknown function DUF444, and one related to *B. subtilis* SpoVR, the function of which is unknown. Proteins of these three families are found together on the chromosome in many other microbial genomes suggesting functional linkage.

Halophiles are known to accumulate gamma-glutamyl-cysteine [43], and two of the signature clusters may be involved in gamma-glutamyl-cysteine metabolism. Cluster 491.1× contains proteins related to glutamate-cysteine ligase, and the *H. volcanii* member of cluster 491.1× was recently shown to have glutamate-cysteine ligase activity [44]. Cluster 1151× includes genes related to glutathione S-transferase, which inactivates toxic compounds by linking them to glutathione. In the halophiles, these may function as gamma-glutamylcysteine S-transferases.

### Habitat-specific clusters

Of the ten haloarchaea with sequenced genomes, four were isolated from water and four were isolated from soil or sediment. The ones isolated from water are *H. walsbyi* [45], *N. pharaonis* [46], *H. marismortui* [47], and *H. borinquense* [48]. *H. mukohataei* and *H. turkmenica* were isolated from saline soils [49], [50], while *H. volcanii* and *H. utahensis* were isolated from lake sediments [51], [52]. The water halophiles and soil/sediment halophiles do not form separate clades in the phylogenetic tree (Figure 1). We looked for clusters present in all water-isolated halophiles that are not present in soil/sediment halophiles and *vice versa*. There were no clusters specific to water halophiles and only three specific to soil/sediment haloarchaea (Table S4). Proteins belonging to two of the soil/sediment-specific clusters (326.1.0× and 2168×) are often found in the vicinity of nucleotide-sugar metabolic enzymes and glycosyl transferases, suggesting they are involved in cell wall biosynthesis [53].

Since there were few protein clusters completely specific to the water or soil/sediment halophiles, we looked for clusters present in three out of four organisms from one group and absent from the other group. There were 16 clusters present in three out of four water halophiles, of which 11 contain hypothetical proteins and four have only general functional annotations (Table S4). The only cluster with a specific annotation is cluster 1816×, formate-tetrahydrofolate ligase.

Of the 26 clusters found in three out of four soil/sediment halophiles and not present in water halophiles, 11 are hypothetical proteins (Table S4). Several of the clusters are involved in polysaccharide degradation. These include a glycosyl hydrolase of family GH4, an alpha-L-arabinofuranosidase of family GH51, a polysaccharide deacetylase and a trehalose utilization protein. Two additional clusters present in three out of four soil/sediment halophiles encode a monooxygenase and an acyltransferase, which are found adjacent to each other on the chromosome and flanked by two IucA/IucC family proteins. These proteins are likely to be involved in siderophore synthesis. *H. borinquense* has three IucA/IucC family proteins but lacks the monooxygenase and acyltransferase, thus it is unclear if it has a complete siderophore biosynthesis pathway.

### All-but-one clusters

We also looked for clusters conserved in all but one genome. These probably indicate recent gene losses in each species. The genomes fell into two groups – those that had 20 or less such clusters and those that had greater than 60 (Table 1). The three genomes that had greater than 60 clusters lost were *H. salinarum*, *H. walsbyi*, and *H. utahensis*. Many of the clusters lost from *H. salinarum* are involved in amino acid synthesis, including genes for the synthesis of glutamate, lysine, ornithine, methionine, and branched chain amino acids. To make up for this, *H. salinarum* does not have more amino acid transporters or secreted proteases than the other haloarchaea, but it is one of only two of the haloarchaea to have a putative peptide symporter of the OPT family (TC 2.A.67). Symporters have low affinity but high capacity, suggesting that *H. salinarum* may prefer to live where there is an ample supply of peptides. Of the clusters not present in *H. walsbyi*, many are involved in flagellum biosynthesis and chemotaxis. However, *H. walsbyi* has a set of gas vesicle proteins to enable motility in the absence of flagella. The clusters in all except *H. utahensis* include several enzymes involved in cobalamin synthesis and several enzymes of biotin utilization and propionate metabolism. *H. utahensis* appears to lack the enzymes for the early steps of cobalamin biosynthesis up to the incorporation of cobalt, but all of the halophiles, including *H. utahensis*, contain the enzymes for the later steps of cobalamin biosynthesis. Biotin and propionate metabolism are discussed further below.

### Central metabolism

Some haloarchaea are known to use the semi-phosphorylated Entner-Doudoroff (ED) pathway for glucose degradation [54], [55], and genes encoding enzymes of this pathway have been identified in several haloarchaea [42]. With the addition of the new genomes, we find that the semi-phosphorylated Entner-Doudoroff pathway is likely to be present in all sequenced haloarchaea except *N. pharaonis*, which does not utilize carbohydrates. Aldolases belonging to two different protein families may be involved. All of the halophiles except *H. salinarum* and *N. pharaonis* have one or more bacterial-type 2-keto-3-deoxy-6-phosphogluconate (KDPGlc) aldolase (COG0800). Also all except *N. pharaonis* have at least one potential aldolase related to the characterized *Sulfolobus* aldolase (COG0329), which is active on KDPGlc and unphosphorylated 2-keto-3-deoxygluconate (KDGlc) [56]. The enzymes of the semi-phosphorylated Entner-Doudoroff pathway are highly conserved in sequence among the haloarchaea, suggesting descent from a common ancestor.

The standard Embden-Meyerhof pathway of glycolysis appears to be incomplete in the halophiles as no 6-phosphofructokinase could be identified. This agrees with previous experimental studies and analysis [42]. Gluconeogenesis is likely to be present in all of the halophile genomes with the possible exception of *H. utahensis*. All except *H. utahensis* have phosphoenolpyruvate (PEP) synthase and/or pyruvate, phosphate dikinase (COG0574). In addition, *H. lacusprofundi* and *H. turkmenica* have ATP-utilizing PEP carboxykinase (COG1866). In *H. utahensis* the only enzyme that potentially can generate PEP for gluconeogenesis is pyruvate kinase. All except *H. utahensis* have a fructose 1,6-bisphosphatase belonging to the same family as the *E. coli* Fbp enzyme (COG0158). All of the halophiles including *H. utahensis* have at least one gene belonging to COG0483, which includes inositol phosphatases and some archaeal fructose 1,6-bisphosphatases [57]. *H. utahensis* has two genes belonging to this family, but they are weakly related to characterized fructose 1,6-bisphosphatases. These findings suggest that *H. utahensis* may lack the gluconeogenesis pathway or have an unusual gluconeogenesis pathway.

Unlike the rest of the archaea, halophiles are thought to use the oxidative pentose phosphate pathway for generation of pentoses [58]. This pathway also generates NADPH for anabolic pathways. All except *H. utahensis* have a probable 6-phosphogluconate dehydrogenase (COG1023), the key enzyme of this pathway. In contrast, *H. utahensis* is the only sequenced haloarchaeon to have transaldolase (Huta_0859) and transketolase (Huta_0860 and Huta_0861), the enzymes of the non-oxidative pentose phosphate pathway. For NADPH generation, *H. utahensis* possesses genes encoding a NAD/NADP transhydrogenase (Huta_2005–2007). None of the other haloarchaea has the genes for this enzyme. The presence of these enzymes only in *H. utahensis* suggests that they may have been acquired through lateral transfer, but phylogenetic analysis was unable to identify the donor (data not shown).

### Nutrient transport

Table 2 presents an overview of nutrient transport in the haloarchaea. All of the haloarchaea have at least five symporters for amino acids and at least two ABC transporters for peptides. Since all except *H. salinarum* can synthesize most or all amino acids, this suggests that amino acids are an important carbon and energy source even in the species that can grow on carbohydrates. All of the haloarchaea also have at least one symporter for nucleosides or nucleobases. Carbohydrate transport is variable. Only half of the halophiles have symporters for sugars, and either none or one ABC transporter for sugars is found in the non-carbohydrate-utilizing organisms. Surprisingly no transporters for sugars could be identified in the *H. utahensis* genome, suggesting that it uses uncharacterized families of sugar transporters.

**Table 2:** Nutrient transport in haloarchaea.

	Sugars		Amino acids		Peptides		Nucleosides/bases	
	Sym	ABC	Sym	ABC	Sym	ABC	Sym	ABC
*H. marismortui*	1	8	7	11	0	3	2	2
*H. salinarum*	0	1	7	0	1	3	1	1
*H. walsbyi*	0	1	5	8	0	4	3	1
*N. pharaonis*	0	0	9	7	0	2	1	0
*H. volcanii*	2	11	11	6	0	13	4	3
*H. lacusprofundi*	0	3	7	5	0	5	2	1
*H. borinquense*	1	5	6	5	0	9	2	1
*H. mukohataei*	1	6	5	2	0	6	1	1
*H. utahensis*	0	0	5	0	0	3	2	0
*H. turkmenica*	4	8	11	7	1	9	5	0

Sym: symporters; ABC: ATP-binding cassette transporters.

There appears to be a connection between some transporters and universal stress protein A (UspA) family proteins. Most amino acid transporters of the amino acid-polyamine-organocation (APC) family are either fused to a UspA domain or adjacent on the chromosome to a UspA protein, and some are both fused and adjacent to UspA family proteins (e.g. Htur_0566). This appears to be specific for the APC family as other potential amino acid symporters of the neurotransmitter∶sodium symporter (NSS) family and the dicarboxylate/amino acid∶cation symporter (DAACS) family are not associated with UspA family proteins. Several transporters of the formate-nitrite transporter (FNT) family are also fused or adjacent to UspA domains (Hmuk_1674, HQ1451A, NP6264A, Hlac_2299, and Htur_2705). The FNT family proteins with associated UspA domains are closely related to each other and to a transporter from *H. marismortui* that lacks a UspA domain (rrnAC0187). They are likely to be formate transporters as the *H. marismortui* and *H. mukohataei* proteins are adjacent to enzymes of folate metabolism. Another transporter with adjacent UspA proteins is a putative acetate transporter of the solute∶sodium symporter (SSS) family. This transporter is found in seven of the ten halophile genomes (e.g. NP5136A), and in all cases is followed by a UspA domain protein. In six of the seven genomes with this transporter, it is close on the chromosome to two acetyl-CoA synthetase genes (e.g. NP5128A and NP5132A). The transporter has highest similarity to subfamily 7 of the SSS family, which includes acetate, propionate, and phenylacetate transporters (see the Transporter Classification Database at www.tcdb.org). UspA family proteins are expressed during many stressful conditions, and they are known to bind ATP, but their exact molecular function is unknown [59]. The UspA domains associated with transporters may play a regulatory role, or may be involved in maintaining transporter function during stressful conditions. A recent report shows that a UspA domain protein is involved in regulation of a transporter [60].

### Secreted proteases

**Figure 2:**
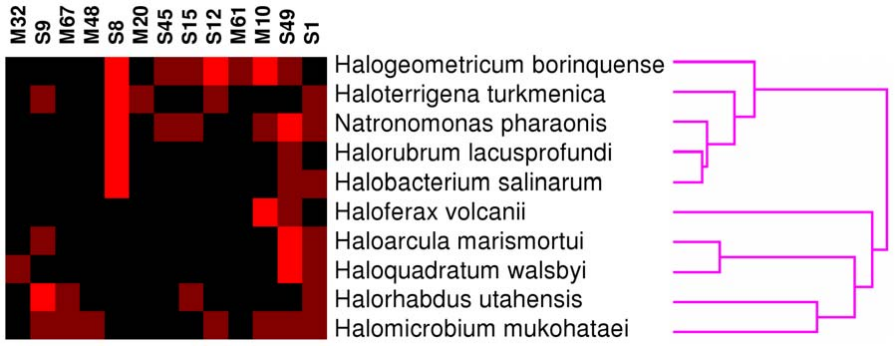
Secreted protease distribution in haloarchaeal genomes. A heat map shows the distribution of MEROPS protease families, and the tree shows the results of hierarchical clustering.

Since the halophiles have numerous transporters for amino acids and peptides, we analyzed the distribution of secreted proteases within their genomes. Only secreted proteases were considered because these are likely to be involved in the utilization of proteins as a nutrient source, while intracellular and integral membrane proteases are involved in a variety of cellular processes. We included proteases that have signal peptides as well as proteins that are likely to be attached to the membrane with the protease domain outside the cell. Signal peptidases (family S26) were excluded from the analysis since they have a specific cellular function. The numbers of secreted proteases in the genomes ranged from 3 to 11. Hierarchical clustering (Figure 2) shows that the halophiles fall into two groups with respect to protease distribution. The main feature separating these groups appears to be the presence or absence of secreted members of protease family S8, which includes subtilisin as well as halolysins from halophilic archaea. The organisms having secreted S8 proteases do not correspond to a habitat-specific or phylogenetic group. The presence of at least three secreted proteases in each genome suggests that all of the halophiles may be capable of degradation of extracellular proteins.

### Amino acid utilization

Since all of the halophiles have at least five amino acid symporters and two peptide ABC transporters, we investigated pathways of amino acid utilization to see if all of the halophiles are capable of using many amino acids. A summary is provided in Table 3. Three degradation pathways were found in all of the ten genomes. All of them had an alanine dehydrogenase similar to the enzyme characterized in *Archaeoglobus fulgidus* [61]. This enzyme could potentially be involved in synthesis of alanine as well as its degradation. All had at least one asparaginase from COG0252 or COG1446. Finally all of them had a pyruvoyl-dependent arginine decarboxylase similar to the enzyme characterized in *Methanocaldococcus jannaschii* [62] and agmatinase. This combination of enzymes produces putrescine and urea from arginine. Additional enzymes of arginine utilization were present in some genomes. Six of the genomes have arginase, which produces ornithine and urea. None of the genomes had ornithine decarboxylase, but all of the ones that have arginase also have ornithine cyclodeaminase which produces proline.

**Table 3:** Amino acid degradation pathways in haloarchaea.

	Ala	Glu	Gly	His	Ile	Asn	Pro	Arg	Thr	Trp
*H. salinarum*	+	+	+	+	+	+	+	+	+	+
*H. marismortui*	+	+	+	+	+	+	+	+		+
*N. pharaonis*	+		+		+	+		+		
*H. walsbyi*	+					+		+		
*H. volcanii*	+		+	+	+	+	+	+	+	+
*H. lacusprofundi*	+		+		+	+	+	+	+	
*H. borinquense*	+		+	+	+	+	+	+	+	+
*H. mukohataei*	+	+	+		+	+	+	+	+	
*H. utahensis*	+					+		+		
*H. turkmenica*	+	+	+	+	+	+	+	+		+

Several other amino acid degradation pathways are found in a subset of the genomes. The glycine cleavage system is found in all genomes except those of *H. walsbyi* and *H. utahensis*. This enzyme complex produces CO_2_, NH_3_, methylene-tetrahydrofolate (THF), and NADH. Methylene-THF has a variety of possible uses within the cell. Another pathway found in all but *H. walsbyi* and *H. utahensis* is isoleucine degradation. A 2-oxoacid dehydrogenase complex involved in isoleucine degradation was recently identified in *H. volcanii* [63], and seven of the other halophiles have genes with at least 68% similarity to the *H. volcanii* genes, indicating that they probably have the same function. Five of the genomes have tryptophanase, which produces pyruvate from tryptophan. A histidine degradation pathway with formiminoglutamate as an intermediate is also found in five of the genomes. Seven have proline dehydrogenase, but only *H. lacusprofundi* has pyrroline-5-carboxylate dehydrogenase to complete proline conversion to glutamate. All of the genomes have threonine dehydratase, but this may be used only for biosynthesis. Five of the genomes in addition have threonine aldolase, which produces glycine and acetaldehyde. Finally, all of the genomes have glutamate dehydrogenase, which may have a biosynthetic role. Four of the genomes have glutamate mutase and methylaspartate ammonia-lyase. These are the first two enzymes of a four-step pathway that produces acetate and pyruvate from glutamate with mesaconate and citramalate as intermediates. However, these two enzymes are likely to be involved in a new pathway for acetate assimilation [64].

Many of the pathways for amino acid degradation are found in a subset of the genomes. They could have been acquired independently by lateral gene transfer or lost in some species. The genes for amino acid degradation in the halophiles are closely related in sequence, suggesting that the common ancestor of haloarchaea was able to degrade many amino acids and that some organisms have lost these pathways.

### Polysaccharide degradation

**Figure 3:**
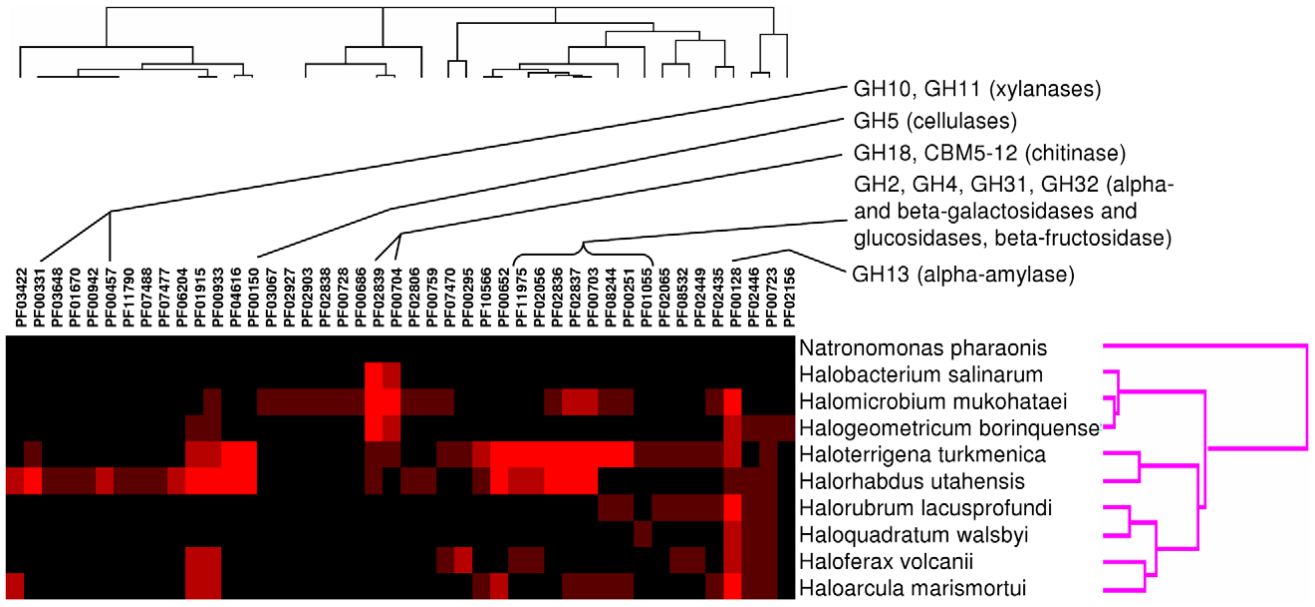
Glycosyl hydrolase distribution in haloarchaeal genomes. The graph shows the results of hierarchical clustering of *Halobacteria* based on the abundance of Pfam domains corresponding to glycosyl hydrolase families and carbohydrate-binding modules (CBMs). Glycosyl hydrolases and CBMs distinguishing groups of *Halobacteria* with different nutritional preferences are highlighted.

According to the distribution of glycosyl hydrolase domains and carbohydrate-binding modules, halophilic archaea can be divided into 3 groups: those that may be capable of degrading plant biomass (*H. utahensis*, *H. turkmenica* and to a lesser extent *H. marismortui* and *H. volcanii*), those harboring family 18 glycosyl hydrolases with possible chitinase activity (*H. salinarum*, *H. mukohataei* and *H. borinquense*) and organisms that are unlikely to degrade any externally provided polysaccharides (*H. lacusprofundi*, *H. walsbyi* and *N. pharaonis*) (Figure 3).

*H. utahensis* and *H. turkmenica* have the two largest sets of proteins with glycosyl hydrolase domains among halophilic archaea (43 and 44, respectively). However, their glycosyl hydrolase complements are markedly different. While *H. utahensis* has five proteins of GH10 family and two proteins of GH11 family, which probably have xylanase activity, *H. turkmenica* has only one GH10 protein and no GH11 members. The abundance of predicted xylanases in the *H. utahensis* genome is in agreement with experimental data that showed xylan-degrading activity of this archaeon [65]. The *H. utahensis* genome contains seven GH5 family proteins and one GH9 family protein (as compared to three and zero in *H. turkmenica* genome). These proteins may have endo-beta-glucanase activity, thus enabling *H. utahensis* to degrade components of the plant cell wall. One of the GH5 proteins in *H. utahensis* (Huta_2387) has been shown experimentally to have cellulolytic activity (T. Zhang et al., in press). *H. utahensis* also has two GH94 proteins that may have cellobiose or cellodextrin phosphorylase activity. On the other hand, the *H. turkmenica* genome encodes four GH32 family proteins predicted to have beta-fructosidase (levanase or invertase) activity that are absent from the *H. utahensis* genome, while both genomes have several GH2 family proteins that may have beta-galactosidase activity.

Three of the genomes from haloarchaea isolated from soil or sediment encode enzymes involved in degradation of pectin. The backbone chains of pectin are made up of either homogalacturonan or rhamnogalacturonan with various side chains, and the main chains are linked together by α-1,5-arabinan chains [66]. *H. turkmenica* has four family 1 polysaccharide lyases (PL) which likely have pectate lyase activity, three of which are close together on the chromosome (Htur_4783, Htur_4785, Htur_4789). Also in the vicinity of these three genes is a family 2 polysaccharide lyase related to pectate lyases (Htur_4786) and two glycosyl hydrolases, one of which may have polygalacturonase activity (Htur_4790). The other *H. turkmenica* PL1 family protein (Htur_4440) is close to one of two pectin methylesterases (Htur_4438) and a rhamnogalacturonan acetylesterase (Htur_4445). *H. turkmenica* also has two family 11 polysaccharide lyases (Htur_3890, Htur_3891) that are highly similar to a *B. subtilis* rhamnogalacturonan lyase and a GH105 protein similar to the RhiN protein of *Dickeya* (formerly *Erwinia*) *chrysanthemi*, which is involved in degradation of rhamnogalacturonate oligosaccharides [67]. *H. utahensis* and *H. volcanii* have much lower capacity for pectin degradation: *H. utahensis* has two probable pectate lyases from family 1, while *H. volcanii* has one pectate lyase and one pectin methylesterase. In addition to enzymes capable of degrading the main chains of pectin, *H. turkmenica*, *H. utahensis*, and *H. volcanii* have GH43, GH51, and GH93 glycosyl hydrolases with similarity to endo- and exo-arabinases that may be capable of degrading the arabinan linking chains of pectin.

### Galactose utilization

*H. lacusprofundi* and *H. mukohataei* have been shown to grow on galactose [49], [68], but the genome sequences suggest that other haloarchaea can also utilize galactose. Also the genome sequences show that two different pathways for galactose metabolism may exist in haloarchaea: the Leloir pathway in *H. utahensis*, and the De Ley-Doudoroff pathway in *H. lacusprofundi*, *H. marismortui*, *H. volcanii*, *H. borinquense*, *H. mukohataei*, and *H. turkmenica*. *H. utahensis* is the only haloarchaeon with genes encoding the three enzymes of the Leloir pathway. No other archaeon possesses a gene for hexose 1-phosphate uridylyltransferase (COG1085, Huta_2170), and *H. volcanii* is the only other haloarchaeon to have a probable galactokinase (HVO_1487). Six haloarchaea (listed above) have genes with high similarity (65–75%) to *E. coli* galactonate dehydratase, one of the enzymes of the De Ley-Doudoroff pathway. Phylogenetic analysis shows that the genes for galactonate dehydratase in the haloarchaea cluster together (data not shown). In the De Ley-Doudoroff pathway, after 2-dehydro-3-deoxygalactonate (KDGal) is formed by galactonate dehydratase, it is phosphorylated by KDGal kinase to form 2-dehydro-3-deoxy-6-phosphogalactonate (KDPGal). KDPGal is then split by KDPGal aldolase to form pyruvate and glyceraldehyde 3-phosphate [69]. None of the halophiles has a gene related to known KDGal kinases (COG3734). KDPGal aldolases belong to the same family as bacterial-type KDPGlc aldolases of the Entner-Doudoroff pathway (COG0800). *H. lacusprofundi* has two proteins related to KDGlc kinase and two proteins related to bacterial-type KDPGlc aldolase. One kinase (Hlac_2870) and aldolase (Hlac_2860) are close on the chromosome to each other and to galactonate dehydratase (Hlac_2866), beta-galactosidase (Hlac_2868), and a probable alpha-galactosidase (Hlac_2869), suggesting that the kinase and aldolase may be involved in the utilization of galactose via the De Ley-Doudoroff pathway. Similarly, *H. volcanii* has three COG0800 proteins, one of which (HVO_A0329) is close on the chromosome to a KDPGlc kinase-related protein (HVO_A0328), galactonate dehydratase (HVO_A0331) and beta-galactosidase (HVO_A0326). Some of the halophiles that have galactonate dehydratase only have one protein related to KDGlc kinase and KDPGlc aldolase. It is possible that in these organisms the proteins are bifunctional, working with both KDGlc and KDGal, similar to the proteins of the *Sulfolobus solfataricus* ED pathway [70].

### Fructose utilization

Fructose can be utilized by some haloarchaea: *H. marismortui*, *H. borinquense*, *H. utahensis*, and *H. turkmenica* have been shown to grow on fructose [48], [50], [52], [71], while *H. mukohataei* has been shown to grow on sucrose [49] and thus will likely also metabolize fructose. The enzyme ketohexokinase was characterized in *Haloarcula vallismortis* but the gene was not identified [72]. *H. marismortui*, *H. volcanii*, and *H. turkmenica* each have one transporter of the phosphotransferase system (PTS), and operon evidence suggests that these are fructose transporters that produce fructose 1-phosphate. The PTS transporters from *H. volcanii* and *H. turkmenica* are close on the chromosome to putative fructose 1-phosphate kinases (COG1105), while in *H. volcanii* the PTS proteins are also close to fructose bisphosphate aldolase. In addition to the three haloarchaea with PTS transporters, *H. utahensis* and *H. mukohataei* also have putative fructose 1-phosphate kinases. In *H. mukohataei* the fructose 1-phosphate kinase (Hmuk_2661) is close on the chromosome to fructose bisphosphate aldolase (Hmuk_2663) and another putative sugar kinase (Hmuk_2662) which may be a ketohexokinase. Surprisingly *H. borinquense* does not have a member of COG1105, despite its known ability to grow on fructose. The protein sequences of all components of the PTS system transporter and fructose 1-phosphate kinase are strongly conserved among the halophiles.

### Xylose utilization

The pathway by which *H. volcanii* utilizes xylose has recently been characterized [73]. The pathway involves formation of xylonate, followed by two dehydratase steps to generate 2-oxoglutarate semialdehyde. This pathway also appears to be present in *H. turkmenica* and *H. lacusprofundi*, both of which were not previously known to utilize xylose. *H. marismortui* is known to produce acid from xylose, and it appears to have 2-dehydro-3-deoxyxylonate dehydratase and xylose dehydrogenase, but it does not have a gene with high similarity to the *H. volcanii* xylonate dehydratase. *H. utahensis* is known to degrade xylan and to be able to grow on xylose [52], [65], and it uses a different pathway for xylose degradation. It has a xylose isomerase (COG2115, Huta_2443) and xylulokinase (TIGR01312, Huta_2446). The resulting D-xylulose 5-phosphate then feeds into the non-oxidative pentose phosphate pathway. *H. utahensis* is the only one of the sequenced haloarchaea to have transaldolase and transketolase of the non-oxidative PPP, which allows it to use this pathway of xylose utilization. *H. borinquense* is known to utilize xylose [48], but it does not have identifiable genes for either of the pathways found in the other haloarchaea. Phylogenetic analysis of xylose isomerase using both neighbor joining (Clustal W) and Bayesian (MrBayes) methods show that *H. utahensis* xylose isomerase branches deeply within Firmicutes with high bootstrap support (not shown), but xylulokinase did not associate closely to any group of organisms.

### Glucuronate utilization

Both *H. utahensis* and *H. turkmenica* have putative glucuronate isomerase (COG1904) and mannonate dehydratase (COG1312), suggesting that they may utilize glucuronate by the same pathway as found in *E. coli* [74]. This pathway produces KDGlc which feeds into the Entner-Doudoroff pathway. *H. utahensis* also has a probable alpha-glucuronidase (Huta_0871) belonging to glycosyl hydrolase family 67, that is adjacent on the chromosome to glucuronate isomerase (Huta_0870) and mannonate dehydratase (Huta_0869). *H. lacusprofundi* has a putative mannonate dehydratase but no glucuronate isomerase, therefore it is unclear whether it has the capacity to break down glucuronate. Since *H. lacusprofundi* is known to grow on mannose [68], it is possible that mannonate dehydratase is used in a pathway for mannose degradation.

### L-arabinose utilization

None of the haloarchaea have been shown to grow on L-arabinose, but the genomes suggest that it may be utilized by some haloarchaea. *H. utahensis*, *H. volcanii*, and *H. turkmenica* all have putative alpha-L-arabinofuranosidases (COG3534). In *H. utahensis* the arabinofuranosidase (Huta_1152) is close on the chromosome to L-arabinose isomerase (Huta_1154) and ribulose 5-phosphate 4-epimerase (Huta_1149), suggesting that *H. utahensis* uses the known bacterial pathway of L-arabinose degradation. A gene similar to ribulokinase was not found in *H. utahensis*, but there is a gene with similarity to xylulokinases (Huta_1150) close to the arabinose degradation genes. Huta_2446 is likely to be a xylulokinase in *H. utahensis* (see above), and Huta_1150 may be a ribulokinase, completing the pathway. This pathway produces D-xylulose 5-phosphate which enters the non-oxidative pentose phosphate pathway. *H. utahensis* is the only haloarchaeon to have the non-oxidative pentose phosphate pathway, which allows it to use this pathway, and it is also the only haloarchaeon to have L-arabinose isomerase.

### N-acetylglucosamine utilization

Since the presence of family 18 glycosyl hydrolases in *H. salinarum*, *H. mukohataei* and *H. borinquense* indicates that they may possess chitinase activity and use chitin as a growth substrate, we attempted to identify enzymes for subsequent degradation of chitooligosaccharides, N-acetyl-glucosamine or glucosamine. We found that *H. mukohataei* likely possesses a beta-N-acetylhexosaminidase (Hmuk_3174) that has 51% similarity to characterized enzymes from *Streptomyces thermoviolaceus* [75] and *Bacillus subtilis* [76]. A chitobiose deacetylase has been identified in *Thermococcus kodakaraensis* belonging to COG2120, which includes other carbohydrate deacetylases [77]. Both *H. mukohataei* and *H. borinquense* have genes belonging to this family, although they are distantly related to the *T. kodakaraensis* enzyme. None of the organisms with family 18 glycosyl hydrolases has been tested for growth on N-acetylglucosamine or glucosamine [48], [49], so the presence of chitinase activity and chitinolytic pathway in haloarchaea needs further experimental elucidation.

### Glycerol metabolism and transport

The haloarchaea encode genes for two different glycerol utilization pathways. All except *N. pharaonis* have a glycerol kinase and glycerol 3-phosphate dehydrogenase, and the genes for both enzymes are found close together on the chromosome. Another pathway involving glycerol dehydrogenase and dihydroxyacetone kinase is present only in *H. lacusprofundi*. *H. volcanii* and *H. walsbyi* have a dihydroxyacetone kinase without glycerol dehydrogenase, and this may be used for metabolism of dihydroxyacetone from the environment [7]. *H. salinarum* encodes a glycerol dehydrogenase but no dihydroxyacetone kinase.

Only *H. mukohataei* has an identifiable glycerol transporter within the genome. It encodes a member of the Major Intrinsic Protein (MIP) family adjacent to glycerol kinase, providing strong evidence for a glycerol transport function. All other haloarchaea that have a glycerol kinase have an uncharacterized membrane protein adjacent (e.g. rrnAC0550), and we predict that these genes encode a new family of glycerol transporters. *H. borinquense* has two glycerol kinases and both have this uncharacterized membrane protein family adjacent to the kinase gene. There are also bacterial homologs of this membrane protein family, and many of them are adjacent to genes involved in glycerol or propanediol metabolism.

### Propionate metabolism

*H. lacusprofundi* is the only halophile that has been shown to grow on propionate, but all of the haloarchaeal genomes, with the exception of *H. utahensis*, contain genes that may encode the methylmalonate pathway for conversion of propionate to succinyl-CoA. All except *H. utahensis* have methylmalonyl-CoA epimerase (TIGR03081) and methylmalonyl-CoA mutase (COG1884 and COG2185). Also they have a biotin carboxylase protein (COG4770) and a carboxyltransferase protein (pfam01039), subunits of a biotin-dependent carboxylase. All except *H. utahensis* also contain a biotin-protein ligase (COG0340) and a BioY family biotin transporter (pfam02632). Propionate or propionyl-CoA may be produced intracellularly from the breakdown of fatty acids, amino acids, or other compounds, or these organisms may be able to use propionate from the environment produced as a result of fermentation.

### Glycine betaine metabolism and transport

Glycine betaine is a compatible solute which is likely to be present in high-salt environments. All of the haloarchaeal genomes except that of *H. mukohataei* encode members of the betaine/carnitine/choline transporter (BCCT) family, which transport glycine betaine and related compounds. Most have one or two members of this family, but *H. turkmenica* has seven. In addition, *H. turkmenica* has an ABC transporter for compatible solutes. Four of the haloarchaeal genomes – *H. marismortui*, *H. walsbyi*, *H. volcanii*, and *H. turkmenica* – encode one or two genes with high similarity to dimethylglycine oxidase from *Arthrobacter globiformis* [78]. In all four genomes these oxidase genes are close on the chromosome to BCCT family transporters. The presence of these enzymes and transporters raises the possibility that some of the halophiles may be able to utilize glycine betaine, dimethylglycine, and/or sarcosine. However, in *H. walsbyi*, betaine was not found to enhance growth [8] and *H. utahensis* could not grow on betaine [52].

## Discussion

### Phylogenetic inference

Our analysis of the ten haloarchaeal genomes provided a phylogenetic tree in which all nodes were well-supported. We here followed the ‘total evidence’ approach for inferring the species tree, which dictates that the best phylogenetic hypothesis is the one based on all available data [79], and that conflict between the species tree and gene trees can at least as well as *via* inferring the gene trees be quantified after combined analysis [34], [35]. Total evidence has been criticized as a ‘verificationist’ approach, particularly by researchers who question the concept of a microbial tree of life (TOL) in general [80]. Other authors counter that concatenated analysis is well rooted in the ‘Popperian principles of background knowledge and corroboration’, and that whether a TOL exists can be assessed, among other means, by checking for high branch support, which would indicate a strong treelike signal, and by comparing previous phylogenetic or taxonomic hypotheses [81]. These issues are of considerable importance for the future of Prokaryote classification [82]. The mostly maximal bootstrap support observed here indicates that *Halobacteriaceae* have evolved in manner consistent with a TOL (Figure 1), which is supported by the majority of the genes (Table S1; Figure S1). Moreover, our supermatrix tree is fully in agreement with the current taxonomy of ingroup and outgroup, whereas considerable conflict was observed in a recent total evidence analysis of *Pasteurellaceae* [83]. The single exception is the branch connecting *H. borinquense* and *H. walsbyi*, which is supported by 700 genes and contradicted by 565 ones, yielding a total positive support of 2,422 steps and a total negative support of −2,366 steps. Considerable incongruence between gene trees may thus be responsible for the low support under MP, but even this branch is maximally supported under ML (Figure 1).

### Incongruence between gene trees and species tree

While our results are in agreement with the optimistic view regarding TOL inference from supermatrices, they nevertheless also indicate that disagreement with the species tree is considerable for some genes (Figures S1 and S2). It is possible to distinguish the effects of artifacts of the phylogenetic inference method, caused e.g. by a low signal-to-noise-ratio, from those of horizontal gene transfer (HGT) because in the former case a correlation between gene length and the extent of disagreement between gene tree and species tree occurs [84]. This correlation is significant in our analysis (Figure S1), but a number of genes appear as outliers of the regression, indicating distinct causes of incongruence such as HGT. The distribution of the median total PBS values over the COG categories reveals that genes related to information processing and storage are more in accordance with the species tree, corresponding to the view that such genes are less frequently horizontally transferred between organisms. However, many exceptions from this rule exist (Table S1; Figure S1), as also observed in a recent analysis of a comprehensive genomic dataset of Prokaryotes [85]. In any case, total evidence analysis in conjunction with partitioned Bremer support [34] apparently worked well in identifying those genes most in disagreement with the species trees (Figure S2). Among the five ‘worst’ gene trees, those for L-lactate permease, Tyrosyl-tRNA synthetase and Cobalamin biosynthesis protein CobN did not comprise any ingroup paralogs in their OrthoMCL clusters; thus, horizontal gene transfer is the most likely explanation for their considerable incongruence with the species tree (Figure S2). HGT between *Candidatus Desulforudis audaxviator* and *Archaea* has been suggested in the literature for CobN [86] and between *Opisthokonta* and *Archaea* for Tyrosyl-tRNA synthetase [87]. In contrast, the OrthoMCL clusters containing either Glucosamine 6-phosphate synthetase or Acyl-coenzyme A synthetase comprised a combination set of inparalogs and possible outparalogs; that is, their disagreement with our species tree estimate is most likely due to a complicated gene duplication/gene loss pattern which may not optimally have been resolved by the OrthoMCL algorithm.

### Soil/sediment vs. water halophiles

While there were few habitat-specific clusters, soil/sediment halophiles were found to have more glycosyl hydrolases as well as genes involved in siderophore synthesis and cell wall metabolism. Some of the halophiles analyzed here were isolated from water (*H. walsbyi*, *N. pharaonis*, *H. marismortui*, *H. borinquense*), while others were isolated from soil (*H. mukohataei*, *H. turkmenica*) or lake sediment (*H. volcanii*, *H. utahensis*). We looked for gene clusters found exclusively in the water halophiles or in the soil/sediment halophiles, but very few clusters were found. There may be several explanations for this finding. One possibility is that the change from water to sediment or *vice versa* has happened several times independently, and different changes in the genome occurred during each round of adaptation. This explanation is supported by the fact that the water and soil/sediment halophiles do not form separate clades in the species phylogenetic tree (Figure 1). Another potential explanation is that the division between water and sediment is not clear-cut. Even though some of these organisms were isolated from water or sediment, they may live part of the time in both environments. Nevertheless, there were some clusters with known functions found in the soil/sediment group and not in the water group. The soil/sediment halophiles tend to have greater numbers of glycosyl hydrolases. Each of the four soil/sediment halophiles has between 15 and 44 glycosyl hydrolases, while the haloarchaea isolated from water have between zero and twelve glycosyl hydrolases. The soil/sediment halophiles also have clusters involved in siderophore synthesis that are missing from the water group. This may be explained by the fact that siderophores are less likely to diffuse away from the producing organism in soil or sediment. Finally the soil/sediment group has two clusters likely to be involved in cell wall metabolism. This may indicate a reaction to the more complex environments that they inhabit.

### Uniqueness of *H. utahensis*

Differences in central metabolism in *H. utahensis* may make it easier for this organism to grow on pentoses. One of the findings from this study is that *H. utahensis* is substantially different from the other halophiles in its central metabolism. It may lack gluconeogenesis, and it appears to use the non-oxidative pentose phosphate pathway and a transhydrogenase instead of the oxidative pentose phosphate pathway used by other haloarchaea. It has the fewest amino acid degradation pathways of any of the sequenced haloarchaea, and thus appears to be the only one of the ten haloarchaea studied here that is specialized only for carbohydrate utilization.

The use of the non-oxidative pentose phosphate pathway may allow for more flexibility in the ability to utilize pentoses as carbon and energy sources. *H. utahensis* is known to degrade xylan and can grow on xylose [52], [65]. The other halophiles have a different xylose degradation pathway that produces 2-oxoglutarate. The xylose and L-arabinose degradation pathways of *H. utahensis* are likely to feed into the pentose phosphate pathway, thus producing fructose 6-phosphate and glyceraldehyde 3-phosphate from phosphorylated pentoses. However, the fate of fructose 6-phosphate is unclear. Halophiles are thought to lack phosphofructokinase, but several including *H. utahensis* have putative fructose 1-phosphate kinase. A possible pathway for fructose utilization would involve the conversion of fructose 6-phosphate to fructose 1-phosphate, followed by phosphorylation to form fructose 1,6-bisphosphate, which would then enter the Embden-Meyerhof pathway. However, further experimental work will be needed to determine how pentoses are metabolized in *H. utahensis*.

### Distribution of catabolic pathways

The distribution of catabolic pathways and the protein sequence conservation of the associated enzymes suggest that the ancestor of haloarchaea could degrade many amino acids but few carbohydrates. The haloarchaea possess pathways for the utilization of amino acids, carbohydrates, and other compounds. The amino acid degradation pathways are found in four to ten of the genomes, and the sequences of the enzymes are closely related, suggesting that they were present in the ancestor of haloarchaea. Three of the genomes have five or fewer amino acid catabolic pathways (*H. walsbyi*, *H. utahensis*, and *N. pharaonis*), while the remaining genomes have at least seven pathways. *H. walsbyi* and *N. pharaonis* also have few glycosyl hydrolases and carbohydrate utilization pathways, and they use very few compounds as energy and carbon sources. *H. utahensis* on the other hand has a large number of glycosyl hydrolases and sugar utilization pathways, showing that it has become specialized for growth on carbohydrates.

In contrast to amino acid utilization pathways, alternative pathways are used for sugar catabolism. For example, *H. utahensis* uses a different xylose degradation pathway than the others, and fructose phosphorylation can occur via the phosphotransferase system or ketohexokinase. In addition, *H. borinquense* is known to grow on fructose and xylose, but it lacks the catabolic enzymes found in other haloarchaea. However, some genes and pathways are highly conserved. The enzymes of the semi-phosphorylated Entner-Doudoroff pathway are closely related in sequence, suggesting that glucose utilization by this pathway was present in the haloarchaeal ancestor. Fructose 1-phosphate kinase is found in five haloarchaeal genomes and is highly conserved. Similarly galactonate dehydratase is found in six of the ten genomes and forms a single phylogenetic group. Overall it appears that the ancestor of haloarchaea was able to use up to ten amino acids as energy and carbon sources, while its ability to use sugars was limited.

### Biotechnological applications

Halophilic glycosyl hydrolases have potential uses in polysaccharide degradation related to biofuel production. An unexpected result from this analysis was the high number of glycosyl hydrolases in two of the organisms. *H. utahensis* has numerous genes related to cellulases and xylanases, while *H. turkmenica* has a large number of predicted pectin-degrading enzymes. Salt-adapted glycosyl hydrolases from haloarchaea may have applications in the depolymerization of plant material for biofuel production. Plant material is pretreated in order to remove lignin and hemicellulose and to reduce the crystallinity of cellulose. Several different methods are used, some of which involve incubation with acid or base [88]. After treatment with these chemicals, a neutralization step is required before further processing, and this produces a salty solution. Salt-adapted glycosyl hydrolases may be useful at this point in the process. Also, treatment of plant material with ionic liquids has been shown to reduce the crystallinity of cellulose and enhance its hydrolysis [89]. One of the glycosyl hydrolase family 5 enzymes of *H. utahensis* has been shown to be a cellulase and to be active in an ionic liquid (T. Zhang et al., in press). Carrying out hydrolysis of lignocellulose under high-salt conditions has the advantage that the possibility of contamination is low.

## Supporting Information

Figure S1 **Sum of partitioned Bremer support over all branches plotted against the number of parsimony-informative characters for all OrthoMCL clusters present in at least four ingroup genomes.** Colors are according to the COG classes: blue, “Information Storage and Processing” (five categories); light blue, “Cellular Processes and Signaling” (ten categories); dark red, “Poorly characterized” (two categories); orange, “Metabolism” (eight categories). The solid line represents a robust-line fit, the dotted line the threshold between overall positive and negative support for the species tree.
(PDF)

Figure S2 **Ingroup-only maximum likelihood (ML) phylogenetic trees inferred from the five OrthoMCL clusters with the most negative overall partitioned Bremer support, i.e., the five genes most in conflict with the species tree (** **Figure 1** **).** The branches are scaled in terms of the expected number of substitutions per site. Numbers above branches are support values from ML (left) and maximum parsimony (MP; right) bootstrapping. Midpoint rooting [90] was applied to all trees. (A) COG1620 (L-lactate permease); (B) COG0449 (Glucosamine 6-phosphate synthetase, contains amidotransferase and phosphosugar isomerase domains); (C) COG1429 (Cobalamin biosynthesis protin CobN and related Mg-chelatases); (D) COG0162 (Tyrosyl-tRNA synthetase); (E) COG0365 (Acyl-coenzyme A synthetases/AMP-[fatty] acid ligases).
(PDF)

Table S1 **Sum of partitioned Bremer support (PBS) values for all OrthoMCL clusters in which at least four ingroup genomes were present, aggregated according to the corresponding COG categories and classes, after correcting for the effect of the number of genomes represented in each cluster and the number of parsimony-informative characters.** Median support is positive for most categories; the fact that most mean support values are negative is most likely due to the presence of few outliers (see Figure S2).
(XLS)

Table S2 **Core clusters. Spectral clusters found in all haloarchaeal genomes.** For each cluster the predicted function and list of locus tags are listed.
(TXT)

Table S3 **Signature clusters.** Spectral clusters found in all haloarchaeal genomes and not found in any other sequenced archaeal genome. The predicted function and locus tags are listed.
(TXT)

Table S4 **Habitat-specific clusters.** Spectral clusters found exclusively in one habitat type and not in the other habitat type. The predicted function and locus tags are listed.
(TXT)

Table S5 **Organisms used in this study and the RefSeq accession numbers for their genome sequences.**
(TXT)
