# Supplementary figures and images for: Novel Insights into the Diversity of Catabolic Metabolism from Ten Haloarchaeal Genomes
Source: PLoS One. 2011 May 25;6(5):e20237. doi: 10.1371/journal.pone.0020237 (PMC3102087; doi:10.1371/journal.pone.0020237)

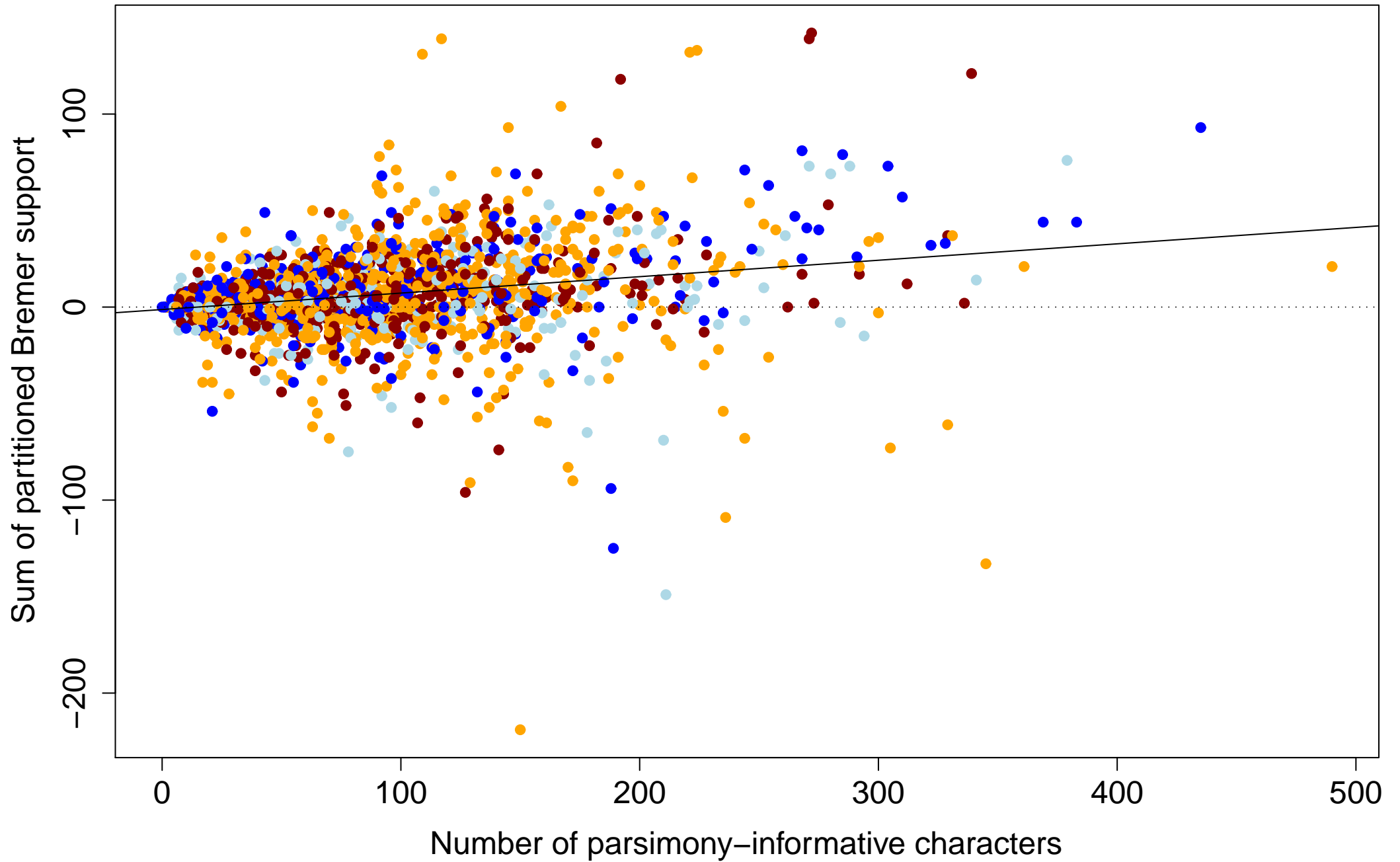

Supplement: Figure S1 — Sum of partitioned Bremer support over all branches plotted against the number of parsimony-informative characters for all OrthoMCL clusters present in at least four ingroup genomes. Colors are according to the COG classes: blue, “Information Storage and Processing” (five categories); light blue, “Cellular Processes and Signaling” (ten categories); dark red, “Poorly characterized” (two categories); orange, “Metabolism” (eight categories). The solid line represents a robust-line fit, the dotted line the threshold between overall positive and negative support for the species tree. (PDF) [file pone.0020237.s001.pdf]

A

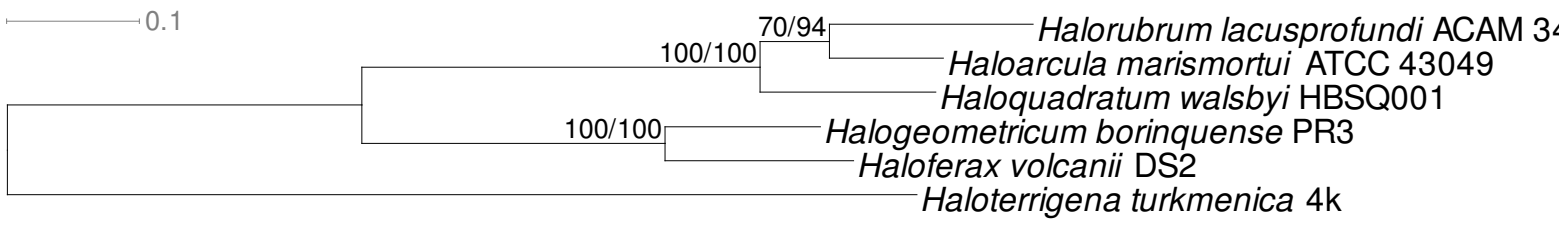

B

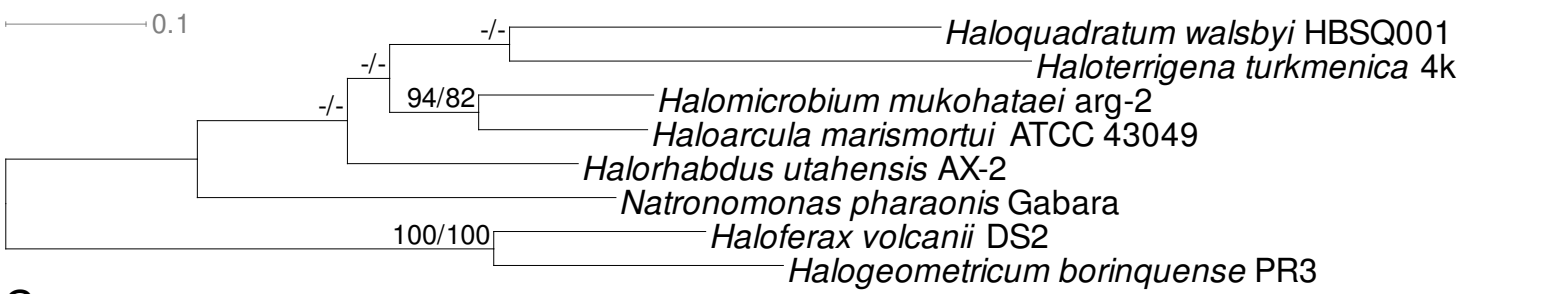

C

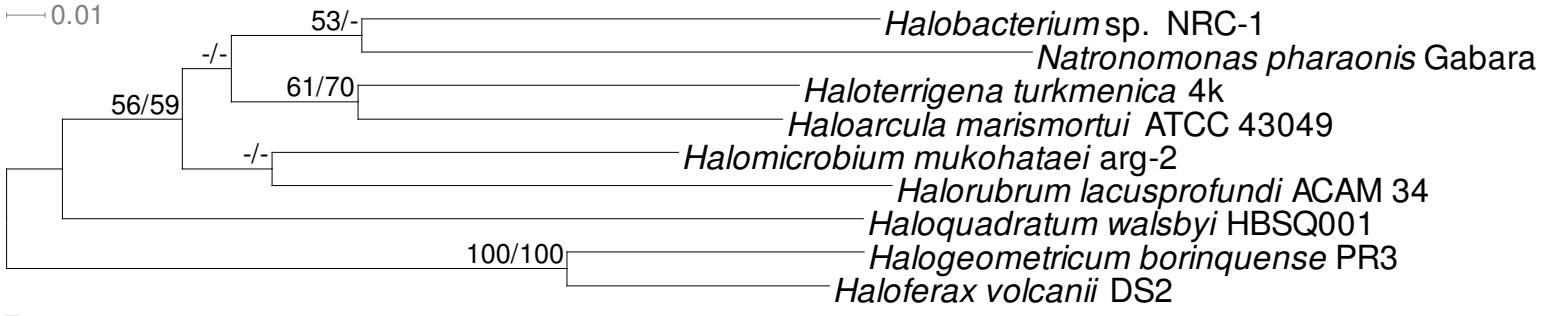

D

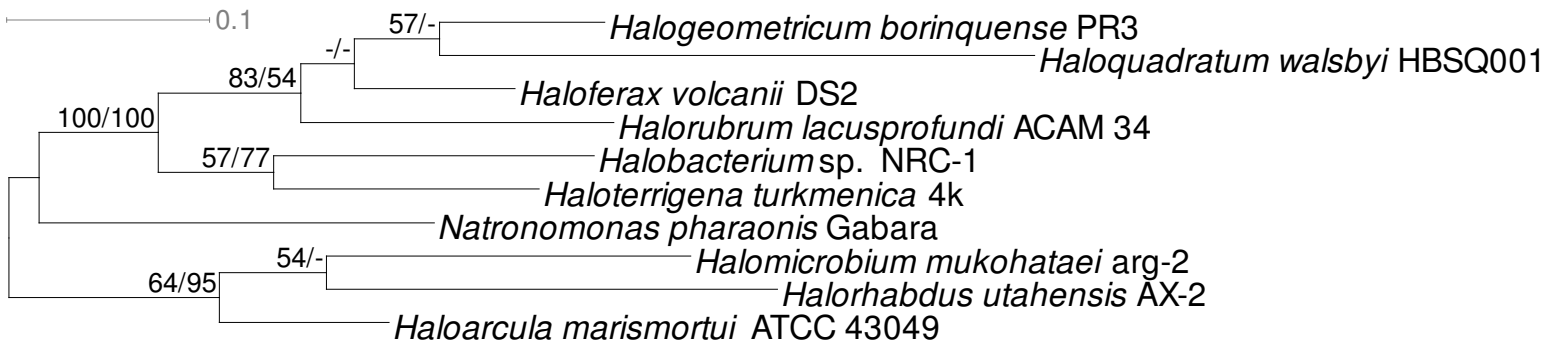

E

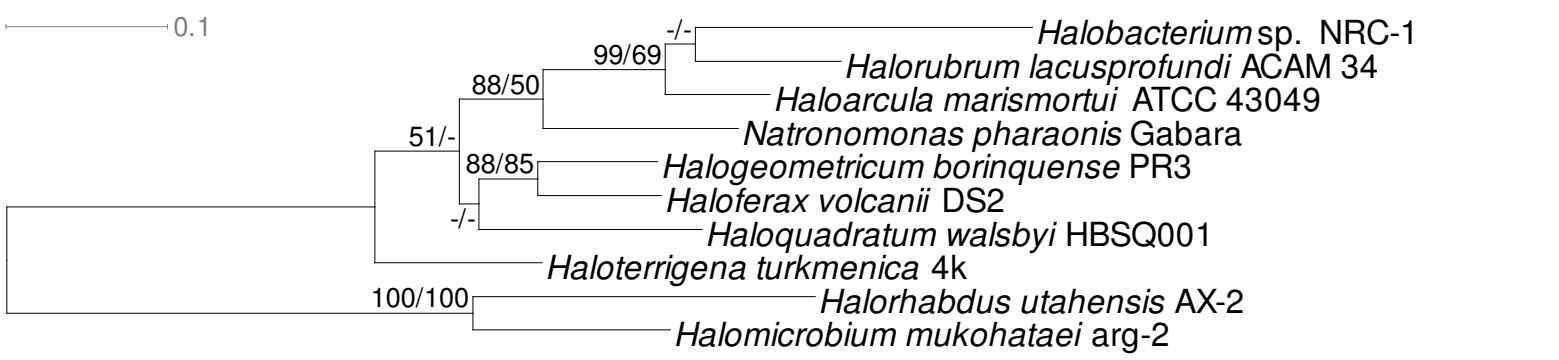

Supplement: Figure S2 — Ingroup-only maximum likelihood (ML) phylogenetic trees inferred from the five OrthoMCL clusters with the most negative overall partitioned Bremer support, i.e., the five genes most in conflict with the species tree ( Figure 1 ). The branches are scaled in terms of the expected number of substitutions per site. Numbers above branches are support values from ML (left) and maximum parsimony (MP; right) bootstrapping. Midpoint rooting [90] was applied to all trees. (A) COG1620 (L-lactate permease); (B) COG0449 (Glucosamine 6-phosphate synthetase, contains amidotransferase and phosphosugar isomerase domains); (C) COG1429 (Cobalamin biosynthesis protin CobN and related Mg-chelatases); (D) COG0162 (Tyrosyl-tRNA synthetase); (E) COG0365 (Acyl-coenzyme A synthetases/AMP-[fatty] acid ligases). (PDF) [file pone.0020237.s002.pdf]
